# Family Decision to Immunize Against Respiratory Syncytial Virus and Associations with Seasonal Influenza and COVID-19 Vaccination

**DOI:** 10.3390/vaccines14010085

**Published:** 2026-01-14

**Authors:** Leah D. Kaye, Benjamin N. Fogel, Ruth E. Gardner, Brody J. Lipsett, Katherine E. Shedlock, Eric W. Schaefer, Ian M. Paul, Steven D. Hicks

**Affiliations:** Public Health Sciences, Penn State College of Medicine, Hershey, PA 17033, USA

**Keywords:** respiratory syncytial virus (RSV), vaccination behavior, vaccination decision, influenza, COVID-19

## Abstract

**Background:** Nirsevimab, a monoclonal antibody for respiratory syncytial virus (RSV), reduces medically attended RSV infections. It was introduced in the 2023–24 RSV season. This study examined the association between caregiver vaccination (seasonal influenza vaccine (SIV), COVID-19, and boosters) and intent to immunize infants against RSV. **Methods:** Data from 118 caregivers with infants ≤ 8 months were analyzed. Chi-squared tests and logistic regression assessed the relationship between caregiver vaccination and intent to immunize against RSV. **Results:** In total, 74.6% of caregivers intended to immunize their infants against RSV. Intent was positively associated with caregiver receipt of a seasonal influenza vaccine (*p* < 0.001), COVID-19 vaccine (*p* < 0.001), and COVID-19 booster (*p* < 0.001). Intent was also associated with older child seasonal vaccination. Caregiver receipt of both COVID-19 vaccinations and boosters had a strong relationship with RSV immunization intent (OR 7.91 (1.90–33.0, *p* = 0.004)). **Conclusions:** Caregiver vaccination behaviors are linked to RSV immunization intent, helping physicians identify hesitant families and prepare for immunization conversations.

## 1. Introduction

Acute bronchiolitis due to respiratory syncytial virus (RSV) is a leading cause of hospitalization in infants under one year old in the United States [1]. Before the 2023–2024 RSV season, palivizumab was the only option for preventing severe RSV disease in infants, but use of this monoclonal antibody is restricted to infants with certain underlying medical conditions or prematurity [2]. Additionally, prevention of severe RSV symptoms with palivizumab requires frequent dosing and is costly [1].

A new monoclonal antibody, nirsevimab, was approved by the Food and Drug Administration (FDA) in 2023 for use during the RSV season. Nirsevimab is a monoclonal antibody that provides temporary passive immunity against RSV. It is approved for a wider range of infants than palivizumab, without requirements for specific underlying conditions. A single administration of nirsevimab prior to the start of an RSV season has been shown to decrease the incidence of medically attended RSV-associated infection in infants [3]. Additionally, RSV pre-fusion (RSVpreF), a vaccination for pregnant patients between 32–36 weeks of pregnancy, was recently approved to help protect newborns from severe RSV infection [4,5]. This vaccination can protect newborns during their first 6 months after birth.

Previous research has examined the relationship between parental vaccine status and decisions to vaccinate children against influenza and severe acute respiratory syndrome coronavirus 2 (SARS-CoV-2, COVID-19) [6,7,8]. For example, parents who receive the seasonal influenza vaccine (SIV) themselves are twice as likely to vaccinate their child against influenza compared to parents who do not receive the SIV [6]. Some studies show that for newer vaccines, such as COVID-19, parents are more hesitant to vaccinate their child than themselves [8]. However, the decision to vaccinate a child against COVID-19 is still associated with parental COVID-19 vaccination status [7]. Other studies have demonstrated a relationship between uptake of SIV and COVID-19 vaccine; if a parent or child receives SIV, it increases the odds that the child will also receive the COVID-19 vaccine [9,10].

Further research indicates that some caregivers perceive “routine” immunizations as those required for school entry differently than seasonal vaccines like SIV and COVID-19 [1,8]. Parents have previously noted concerns regarding co-administration of SIV and routine vaccinations, including general safety, risk of adverse effects, children being too young to receive SIV, or distrust of medical professionals [1,11]. These concerns have affected their decision to vaccinate against seasonal influenza [1].

With the release of nirsevimab during the 2023–2024 RSV season, we sought to describe the factors that influence caregivers’ decisions to immunize their infants against RSV, as reported uptake of the vaccine has been variable [12,13]. We examined whether there is an association between parental receipt of SIV or COVID-19 vaccination and immunization of their infant with nirsevimab. We also assessed the association between parental choice to vaccinate other children in their home against seasonal influenza or COVID-19 and the choice to immunize their infant with nirsevimab. We hypothesized that caregiver choice to vaccinate either themselves or their other children with these vaccines would have a positive association with immunizing their infant with nirsevimab. We hope that these findings will help pediatricians better prepare for RSV immunization discussions with their patients.

## 2. Materials and Methods

**Study Population:** This study included a convenience sample of 169 caregivers with an infant ≤ 8 months of age, receiving care at Penn State Health Children’s Hospital in south-central Pennsylvania and 3 associated Penn State Health primary care pediatric practices between November 2023 and March 2024. The age of ≤8 months was chosen to match the recommended age for this immunization during the season it was released. Exclusion criteria included infant gestational age < 35 weeks, infant age > 8 months at the time of survey completion, surveys with less than 50% of completed items, and duplicate surveys. Enrollment occurred in the newborn nursery and pediatric primary care offices affiliated with our academic medical center. A total of 51 survey responses were excluded due to child age > 8 months or otherwise ineligible (n = 24), incomplete survey responses (n = 17), and duplicate survey responses (n = 10). The final analysis contained 118 responses. COVID-19 and SIV vaccination status for caregivers and siblings were collected by self-report.

The primary medical outcomes were nirsevimab “uptake” and “intent.” Uptake was defined as the child receiving nirsevimab or the biological mother receiving RSV immunization while pregnant (negating the need for infant receipt). Intent was defined as uptake of RSV protection (maternal vaccine or infant nirsevimab) plus the additional caregivers who wanted their child to receive nirsevimab but could not because it was temporarily out of stock (low availability from manufacturer shortage). Intent was included as an outcome because it best reflects caregiver choice to immunize against RSV disease in a perfect setting (i.e., one without the supply shortages in 2023–2024).

**Data Collection:** Recruitment and data collection were completed by providing eligible participants with a paper flyer containing study information and a unique QR code link to a 57-item survey for self-administration. This flyer was provided in the inpatient newborn nursery setting and at outpatient well visits (newborn and 1, 2, 4, and 6 months). A monetary incentive was provided for study completion. Participation was limited to one per household to avoid duplicate responses. Each participating family completed an electronic survey at the time of enrollment. The survey instrument was developed by the study team based on the prior literature examining parental vaccine decision-making and was refined through review by primary care clinicians. Survey domains included caregiver demographics, healthcare exposure, infant clinical characteristics, awareness of RSV and available preventive strategies, caregiver and sibling vaccination history for influenza and COVID-19, and intent regarding RSV immunization. The survey was available in English, Spanish, and Nepali, consistent with the primary languages of the majority of the local patient population.

Survey questions inquired about demographic information, such as gender, primary language, race, ethnicity, number of children and adults in the home, highest completed education level, household annual income bracket, marital status, healthcare worker status, health insurance type, and plans for daycare. Survey questions also inquired about family immunization history, such as if the mother received the maternal RSV vaccine during pregnancy, if the responder receives annual influenza vaccines for themselves, if the responder received any COVID-19 vaccines, if the responder plans to receive a yearly COVID-19 booster, if other children in the home receive annual influenza vaccines, and if other children in the home have received any COVID-19 vaccines.

**Statistical Analysis:** De-identified data were collected in REDCap. Descriptive statistics were used to summarize the demographic characteristics of the study population. Chi-squared tests were used to examine the association of intent to immunize against RSV (“intent”) with the receipt of other vaccines, “receipt of SIV for adults,” “receipt of initial COVID-19 vaccine for adults,” “receipt of COVID-19 boosters for adults,” “receipt of SIV for older children,” and “receipt of COVID-19 vaccine for older children.” Next, logistic regression models were used to examine influenza and COVID-19 vaccination choices simultaneously in regard to the outcome of RSV immunization. We fit this model only for adult SIV and COVID-19/COVID-19 booster receipt because only 67% of respondents had older children.

## 3. Results

Of the 169 caregivers who completed the survey, 118 met all inclusion/exclusion criteria and were used in the analysis. In total, 658 infants of less than 8 months were seen in the clinics during the study time, and 344 infants were admitted to the newborn nursery; the response rate was 16.9%. The majority of respondents were mothers (n = 109, 92.4%). Most (n = 111, 94.9%) did not complete the language spoken at home section. The majority (n = 112, 96.6%) of the adult respondents identified as not Hispanic/Latino. Just over half (n = 79, 67.5%) of parents had private insurance, while 23.1% (n = 27) had Medicaid, with the remainder answering “none,” “self-pay,” or “other.” Over half completed at least college-level education, with the least common educational level being “some high school” or “completed trade school” (n = 4 for each, 3.4%) and the most common being post-graduate degree (n = 42, 35.6%). Approximately half (n = 66, 55.9%) of respondents or their partner worked in healthcare.

The majority of adult respondents had heard of RSV (n = 112, 95.7%) and were aware of an immunization available to prevent RSV in young children (n = 95, 81.2%). Most respondents indicated there were no high-risk household members regarding RSV disease (n = 99, 83.9%). Slightly over a third of respondents indicated that the biological mother of the child received the maternal RSV vaccine during pregnancy (n = 42, 35.6%). A further descriptive summary of variables can be found in Table 1.

Most caregivers reported receiving SIV every year or most years. Some of the original variables (“No, never,” “Yes, occasionally,” “Yes, most years,” and “Yes, every year”) were collapsed together for clarity when examining responses regarding SIV and COVID-19 vaccination. We made the decision to group “No, never” and “Yes, occasionally” as “No” vs. “Yes, most years” and “Yes, every year” as “Yes” as this seemed to most accurately reflect the general behavior of a respondent in an average year.

Most received an initial COVID-19 vaccine, but less than half reported regular COVID-19 boosters (every year or most years). Of the 80 infants in this study who had older children in the household, most of the older children received SIV every year or most years. Less than half of the older children received any COVID-19 vaccine (initial series or boosters). A descriptive summary of these variables can be found in Table 2.

The majority of infants (n = 79, 66.9%) received protection from RSV with either nirsevimab or maternal vaccine. A slightly larger proportion of caregivers expressed overall intent to immunize (n = 88, 74.6%). We examined the outcome of “uptake,” meaning that the child received RSV immunization; this was 66.9% (n = 79) of respondents. We also examined the outcome “intent”, meaning those whose mother received RSV immunization while pregnant (thus negating the need for infant receipt), those whose child received the immunization, and those who intended to obtain the vaccine but could not due to the unavailability of the product in the clinic. “Intent” represented 74.6% (n = 88) of respondents. We primarily discuss the “intent” cohort results in this section, as this best reflects caregiver choice to protect their infant from RSV disease in a perfect setting, i.e., one without the shortages in supply that occurred this year.

Caregivers’ intent to immunize their infant against RSV was associated with yearly caregiver receipt of SIV (*p* < 0.001) and caregiver receipt of COVID-19 vaccine and vaccine boosters (*p* < 0.001). Caregiver intent to immunize their infant against RSV was also associated with yearly receipt of SIV or any COVID-19 vaccination for older siblings in the household (Table 3, N = 80, as only 80 of the 118 respondents had older children).

A logistic regression model examined caregiver vaccination choices around SIV and COVID-19 simultaneously, relative to infant RSV immunization intent. This was performed only for caregiver SIV and COVID-19 vaccines, rather than for siblings as well, due to only 32% of respondents having older children in the household. In order to do this, we created a new variable, “initial COVID-19 vaccine and boosters”, to allow us to create regression models that include flu vaccine, COVID-19 vaccine, and COVID-19 vaccine and boosters regarding the outcome of RSV vaccine receipt. This new variable creation was necessary due to the systematic relationship between COVID-19 vaccines and COVID19 boosters, as one cannot receive a COVID-19 booster if one has not yet received a COVID19 vaccine. Examining this model, initial COVID-19 vaccine and booster receipt (n = 53, 44.9% of sample) had a significant association with intent to immunize against RSV (OR 7.91 (1.90–33.0, *p* = 0.0004). The results are shown in Table 4.

## 4. Discussion

Our study demonstrated a significant relationship between adults who vaccinate themselves against influenza and/or COVID-19 and intent to immunize their child against RSV. We also found a positive relationship between caregiver choice to immunize their other child(ren) against influenza and COVID-19 and the choice to immunize their infant against RSV. The strongest association in our study showed that adults who receive all COVID-19 vaccinations recommended by ACIP through 2024 are most likely to immunize their infant against RSV. One potential explanation for these findings is that caregivers who routinely receive seasonal and updated vaccines may demonstrate greater trust in public health guidance and greater comfort with newer immunization strategies. This may be particularly relevant for nirsevimab, which differs from traditional vaccines in both mechanism and messaging.

These results add to data from prior studies indicating that adult vaccination status has a positive association with a caregiver’s intent to immunize their child, previously seen with decisions regarding influenza and COVID-19 vaccination [3,6,8,9]. From a clinical perspective, these findings suggest that routine assessment of caregiver influenza and COVID-19 vaccination status may serve as a pragmatic screening tool to identify families who may benefit from anticipatory counseling regarding RSV immunization. Identifying families more likely to be vaccine-hesitant toward nirsevimab may allow providers to prepare for more in-depth discussions regarding immunization, such as its safety and efficacy, or how it differs from other immunizations that have been declined by the family. Additionally, it could allow for a “bundled” approach for families, acknowledging their past behaviors toward protection against other respiratory viruses and promoting protection against RSV.

Limitations of this study include the small sample size, which limits the power of the study; future studies would provide greater insight if they were able to collect a larger sample size. The response rate overall was low, and most respondents in the study were mothers, making it harder to generalize this study to immunization choices of non-mother caregivers.

Another challenge in this study included a number of demographic limitations to generalizability. A substantial percentage of respondents work in healthcare or have a partner in healthcare; it is likely that the attitudes of the general public toward immunization differ from those of people who work in a healthcare setting. A caregiver or partner working in healthcare has been shown to be associated with RSV immunization intent [14]. Some healthcare settings mandate influenza vaccination, which may have led to an increased number of adult respondents who receive yearly flu vaccines as compared to the general public.

Our survey sample also included a high rate of higher education (over half of the respondents), which limits generalizability to other settings. Future investigations regarding this issue in our clinic could be balanced by seeking out a cohort of non-healthcare workers or a cohort without higher levels of education.

The majority of respondents were non-Latino. We were unable to collect data regarding the demographics of the non-respondents to account for potential selection bias in this area. Previous research has recognized ethnicity as one of the factors associated with vaccination disparities [15], so the absence of ethnic diversity in our sample size limits its generalizability.

A large percentage of respondents had private insurance, which is representative of our clinic setting (approximately 60% private insurance) but not representative of all clinics nationwide. Private insurance is independently associated with intention to immunize against RSV [14,16,17] and with decisions around SIV [18]. These represent potential confounders when analyzing the relationship between SIV, COVID-19, and immunization against RSV. We did not independently account for these factors in this analysis.

The need to designate “intent to immunize” for situations where nirsevimab was unavailable is also a limitation, as it relies on the assumption that all who intended to immunize would have done so had the immunization been available at the time of response. Not all infants seen in the clinic were born within our medical system, and not all mothers had prenatal care within our system; multiple independent hospital systems exist in our region of the United States. They do not all share a universal electronic medical record; therefore, maternal receipt of Abrysvo could not be independently confirmed. This need to rely on parental self-reporting is also a limitation.

## 5. Conclusions

Caregiver engagement with seasonal influenza and COVID-19 vaccination was strongly associated with intent to immunize infants against RSV during the first season of nirsevimab availability. These findings highlight the importance of considering caregiver vaccination behaviors when designing clinical workflows and public health strategies to support RSV prevention.

## Figures and Tables

**Table 1:** Descriptive summary of responses.

	Total N = 118 n (%)
Mother	109 (92.4)
Not Hispanic/Latino (N = 116, 2 responses missing)	112 (96.6)
Insurance Type	
Private	79 (67.5)
Medicaid	27 (23.1)
Self-pay, other, none, or missing	12 (10.1)
Education Level	
Post-graduate degree	42 (35.6)
Completed college	33 (28.0)
Completed trade school	4 (3.4)
Some college	15 (12.7)
High school graduate	20 (16.9)
Some high school	4 (3.4)
Language spoken in home (1 response missing)	
English	111 (94.9)
Spanish	2 (1.7)
Nepali	1 (0.9)
Other	3 (2.6)
Caregiver or partner in healthcare	66 (55.9)
No plans for daycare	78 (66.1)
Child never had NICU hospitalization	99 (84.6)
Caregiver does not smoke	110 (94.0)
Caregiver has heard of RSV (N = 117, 1 response missing)	112 (95.7)
Caregiver is aware of new RSV immunization (N = 117, 1 response missing)	95 (81.2)

**Table 2:** Descriptive summary of vaccine variables.

	Total (N = 118) n (%)
Caregiver receives yearly SIV *	82 (69.5)
Caregiver receives any COVID-19 vaccine	88 (74.6)
Caregiver receives COVID-19 boosters *	53 (44.9)
Other children > 6 months old at home	80 (67.7)
Older children receive yearly SIV (N = 80) *	56 (70.0)
Older children receive any COVID-19 vaccine (N = 80 with older children)	33 (41.3)

* Every year or most years was considered a positive response for these items.

**Table 3:** Association between caregiver/sibling vaccination and intent to immunize infant against RSV.

Vaccination Status	Caregiver Intends to Immunize Against RSV N (%)	*p*-Value
Caregiver receives yearly SIV * [N = 118]		<0.001
Yes, every year or most years	70/82 (85.4%)	
No, never or occasionally	18/36 (50.0%)	
Caregiver received any COVID-19 vaccine [N = 118]		<0.001
Yes	74/88 (84.1%)	
No	14/30 (46.7%)	
Caregiver receives COVID-19 boosters * [N = 118]		<0.001
Yes, every year or most years	49/53 (92.5%)	
No, never or occasionally	39/65 (60.0%)	
Older children receive yearly SIV [N = 80 with older children] *		0.002
Yes	47/56 (83.9%)	
No	12/24 (50.0%)	
Older children receive any COVID-19 vaccine [N = 80 with older children]		<0.001
Yes	31/33 (93.9%)	
No	28/47 (59.6%)	

* Every year or most years was considered a positive response for these items.

**Table 4:** Logistic regression model examining association between caregiver vaccine status and infant RSV immunization intent.

	OR (95% CI)	*p*-Value
Caregiver receives SIV *		
Every year or most years	2.39 (0.82–6.96)	0.11
Never or occasionally (reference)		
Caregiver COVID-19 vaccine experience		
None (reference)		
Received initial COVID-19 vaccine, no boosters	2.05 (0.67–6.22)	0.21
Received initial COVID vaccine and boosters *	7.91(1.90–33.0)	0.004

* Every year or most years was considered a positive response for these items.

## Data Availability

The raw data supporting the conclusions of this article will be made available by the authors upon request.
